# Pharyngeal pumping rate does not reflect lifespan extension in *C. elegans* transgenerational longevity mutants

**DOI:** 10.17912/micropub.biology.000719

**Published:** 2023-01-27

**Authors:** Jaime C. Croft, Arthur Colunga, Lea Solh, Michaela K. Dillon, Teresa Wei-sy Lee

**Affiliations:** 1 Department of Biological Sciences, University of Massachusetts Lowell, MA, USA

## Abstract

Epigenetic modifications must be reprogrammed with each new generation. In *Caenorhabditis elegans* , defects in histone methylation reprogramming allow for the transgenerational acquisition of longevity. For example, mutations in the putative H3K9 demethylase JHDM-1 extend lifespan after six to ten generations. We noticed that long-lived *jhdm-1* mutants appear healthier than wild-type animals from the same generation. To quantify health, we compared the common metric of pharyngeal pumping rate at specific adult ages between early-gen populations with normal lifespans and late-gen populations with long lifespans. Longevity did not affect pumping rate, but long-lived mutants stop pumping at a younger age, suggesting a possible conservation of energy to extend lifespan.

**Figure 1. Lifespan extension in jhdm-1 mutants does not affect pumping rate, but long-lived populations stop pumping at a younger age.**
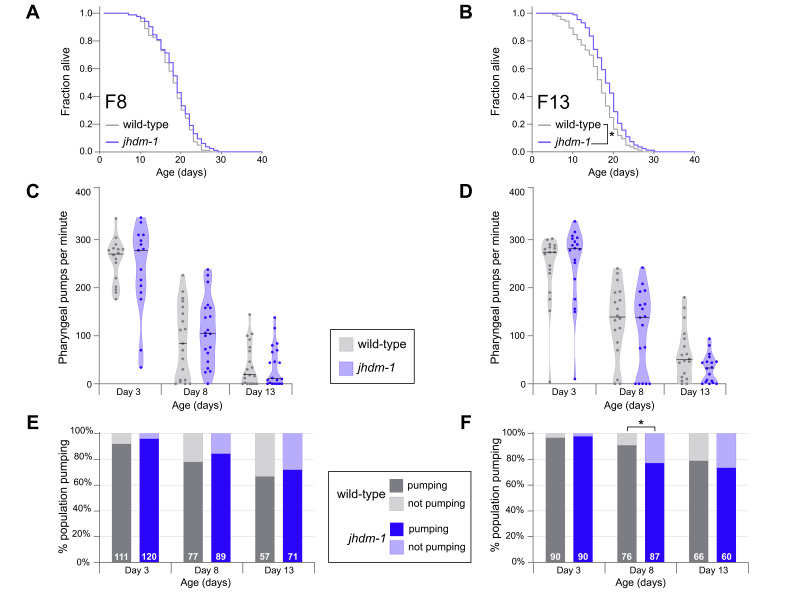
**(A, B)** Representative lifespan of *jhdm-1 ( ok2364 )* mutants (blue) and generation-matched wild-type animals (gray) in early-gen ( **A, C, E** ) and late-gen populations ( **B, D, F** ). Lifespan was assessed for 3-4 independent transgenerational replicates, with one representative replicate shown here. **(A)** In early-gen F8 populations, *jhdm-1* mutants are not long-lived. **(B)** In late-gen F13 populations, the median lifespan of *jhdm-1* mutants was 5.26% longer than generation matched wild-type *p<0.05 with a long-rank test. **(C, D)** Pharyngeal pumping rate of *jhdm-1* mutants (blue) in both early-gen ( **C** ) and late-gen ( **D** ) populations did not differ from generation matched wild-type animals (gray). Graphs combine data from three independent transgenerational replicates. Median is shown as a black line. **(E, F)** Percentage of the population pumping in *jhdm-1* mutants (blue) and generation-matched wild-type (gray) across generational time. Graphs combine data from two independent transgenerational replicates. Total N values are shown in white. **(E)** Throughout adulthood, the percentage of early-gen *jhdm-1* mutants that are pumping does not differ from wild-type. **(F)** In populations of late-gen *jhdm-1* mutants that have acquired longevity, fewer animals are pumping in middle age (Day 8 of adulthood). *p<0.05 with a Fisher’s exact test.

## Description

Epigenetics refers to the inheritance of molecules other than DNA that can transmit information about a parent’s environment without making permanent changes to genomic sequence (Heard and Martienssen 2014; Perez and Lehner 2019; Cavalli and Heard 2019). The provisional nature of epigenetic inheritance provides subsequent generations with an “epigenetic memory” of previous conditions that might impact their survival and that of subsequent generations. However, this memory can be maladaptive if maintained beyond its relevancy, which is why epigenetic inheritance is often intergenerational: limited to being passed from parent to child (Burton and Greer 2022). Inheritance that lasts for three or more generations is considered transgenerational, because epigenetic memory is passed to an individual that had no cells present in their ancestor at the initiation of that memory.

One of the molecules capable of transmitting epigenetic memory is chromatin, which consists of DNA wrapped around histone proteins to form nucleosomes that determine DNA accessibility. Post-translational modifications can be added and removed from histone tails, which alter nucleosome positioning and regulate gene expression. For example, studies in the nematode *Caenorhabditis elegans* have shown that histone modifications are capable of mediating the transgenerational inheritance of complex traits over three or more generations, including increased stress resistance (Kishimoto *et al.* 2017; Wan *et al.* 2021), avoidance of pathogens (Moore *et al.* 2019; Kaletsky *et al.* 2020), and extended lifespan (Greer *et al.* 2011). Furthermore, mutations in histone modifiers allow for the gradual transgenerational acquisition of traits like sterility (Katz *et al.* 2009) and longevity (Greer *et al.* 2016; Lee *et al.* 2019).

The addition of methyl groups to lysine 9 of histone H3 (H3K9me) is commonly associated with gene repression. In *C. elegans* , H3K9me2 is the modification most associated with heterochromatin factors (McMurchy *et al.* 2017). JHDM-1 is a putative demethylase for H3K9, and a homolog of *Schizosaccharomyces pombe* EPE-1, the H3K9 demethylase required for the inheritance of H3K9me across many cell divisions (Rangunathan *et al.* 2015; Audergon *et al.* 2015). Previously, we have shown that *jhdm-1* mutants accumulate repressive H3K9me2 over multiple generations, with levels significantly higher than wild-type by generation F12 (Lee *et al.* 2019). Correspondingly, *jhdm-1* mutants experience up to a 30% increase in lifespan, usually seen by six to ten generations after recovery from a thaw. Based on these prior observations, mutant populations with normal levels of H3K9me2 and a wild-type lifespan are considered early-gen (F1 to F8), whereas mutant populations with high levels of H3K9me2 and extended lifespans are considered late-gen (F8 and beyond, classified based on the appearance of longevity in the population).

When characterizing longevity, we noticed that late-gen *jhdm-1* mutants appeared healthier than their wild-type counterparts – middle-aged and elderly mutants moved more robustly, and their physiology seemed better maintained. Therefore, we began quantifying metrics that correlate with organismal health. Health has been evaluated in other *C. elegans* lifespan mutants with extended lifespans (Bansal *et al.* 2015; Hahm *et al.* 2015) and shortened lifespans (Rollins *et al.* 2017). However, previous studies were performed in mutants where lifespan is immediately affected in a single generation. Additionally, these studies differed on which health metrics were best correlated with changes in lifespan, including stress resistance, motility, and pharyngeal pumping. For example, several long-lived mutants appeared to extend lifespan by increasing the period of frailty at the end of life, rather than proportionally increasing all life stages.

To see whether health metrics capture the transgenerational acquisition of longevity in genetically identical populations, we examined the commonly-used metric of pharyngeal pumping. *C. elegans* ingests food through the pharynx, a neuromuscular pump. Measuring pumping rate is a simple way to assess food intake and the functional decline of a cardiac-like muscle (Huang *et al.* 2004; Chow *et al.* 2006; Bansal *et al.* 2015). We assessed lifespan and pumping rate in early-gen and late-gen populations. To capture changes over the full healthspan, we quantified pumping on days 3, 8, and 13 of adulthood. Early-gen *jhdm-1* mutants have normal lifespans compared to wild-type populations (Fig. 1A and Lee *et al.* 2019), and experience no difference in pumping rate from wild-type populations at any of the timepoints examined (Fig. 1C) – all animals experience a similar reduction in pumping rate as they aged, consistent with prior reports. Similarly, late-gen *jhdm-1* mutants that had acquired longevity (Fig. 1B and Lee *et al.* 2019) also have no difference in pumping rate (Fig. 1D) – these long-lived *jhdm-1* mutants experience a reduction in pumping as they age, similar to their wild-type counterparts. Therefore, pumping rate does not effectively capture the qualitative changes in health we observed in transgenerational *jhdm-1* mutants. This finding is in agreement with some prior studies that characterize pharyngeal pumping as a metric of absolute age (number of days alive), rather than health or apparent age (where healthier individuals appear younger) (Bansal *et al.* 2015; Rollins *et al.* 2017).

Because pumping rate did not differ between normal-lived and long-lived *jhdm-1* mutant populations, we next examined the percentage of the population exhibiting any pharyngeal pumping throughout their lifespan. Work by Huang and colleagues used this metric in addition to pumping rate to evaluate the health of long-lived mutant populations and found significant differences in percent pumping between certain long-lived mutants and wild-type (Huang *et al.* 2004). In early-gen populations, there is no difference between *jhdm-1* mutants and wild-type animals at any timepoint (Fig. 1E). However, in long-lived late-gen *jhdm-1* mutants, fewer middle-aged adults were pumping on Day 8 compared to wild-type (Fig. 1F, p = 0.021, from two independent transgenerational replicates). However, by Day 13, when animals are elderly, both *jhdm-1* mutants and wild-type animals experience the same decline in percentage pumping. Therefore, we find that percentage pumping is a metric that correlates better with our observed extension of lifespan than the rate of pumping, with the most informative timepoint occurring at Day 8 of adulthood in middle-age.

In this study, we have identified one metric that captures the changes in health observed in *jhdm-1* mutants after populations have acquired longevity. It is important to note that we do not observe a change in percentage pumping across the lifespan – we only see a decrease in middle-aged *jhdm-1* mutants, which is the age at which we first start seeing death in wild-type populations. Surprisingly, we found that fewer long-lived *jhdm-1* mutants are pumping. This result was unexpected – because these populations live longer, we hypothesized that they might pump for longer as well. These data might indicate that long-lived *jhdm-1* mutants experience the largest decrease in percentage of the population pumping between young adulthood (Day 3) and middle age (Day 8). It is possible that an initial decrease in pumping could extend the period of time spent pumping, and may even contribute to longevity. For example, one theory of aging proposes that the energy expended within a single lifetime is a finite resource – therefore, conserving energy in young individuals could ultimately extend lifespan (Escala 2022). Future studies will assess additional health metrics in transgenerational *jhdm-1* mutants to establish a well-rounded understanding of how health is coupled to lifespan.

## Methods

***C. elegans* husbandry:** All *C. elegans* strains were cultured at 20° C on 6-cm nematode growth medium (NGM) agar plates seeded with OP50 strain *E. coli* grown in Luria Broth (LB). Each independent thaw of a strain is considered a separate transgenerational replicate, with the P0 designated as the progeny of L1s who survived the thaw. To maintain a transgenerational population, three L4 hermaphrodites were transferred every fourth day from the previous population, to prevent populations from becoming crowded or starved. All experiments were performed with age-matched and generation-matched populations.

**Longevity:** To start each assay, young adults (on their first day of egg laying) were allowed to lay embryos for 4-6 hours to hatch a synchronized population. Hermaphrodites were transferred to new plates at the L4 stage: for longevity assays, 90 animals per condition were transferred with 30 animals on each plate (with an additional 90 animals set aside to use for the pharyngeal pumping assay). Animals were transferred every day or every other day during their fertile period (usually the first ten days). Plates were scored for survival once a day. Animals that did not respond to repeated gentle strokes with a platinum pick were scored dead. Animals that died via dessication, ruptured vulvas, or matricide were censored from the experiment. Kaplan-Meier survival curves were generated in GraphPad Prism 9 and significance was calculated between generation-matched populations using a log-rank test (Mantel-Cox).

**Pharyngeal pumping rate:** As described above, 90 L4-stage hermaphrodites per condition were transferred to new plates with 30 animals on each plate (separate from the animals used for the longevity assay). Animals were transferred every day or every other day during their fertile period (usually the first ten days), and scored for pumping on Days 3, 8, and 13 of adulthood. Pharyngeal pumping rate was collected at each timepoint by using LabScope (Zeiss) to record an animal’s pharynx for 60 seconds at 50x magnification with an Axiocam 208 Color Microscope Camera (Zeiss) mounted to a Stemi 508 Stereo Microscope (Zeiss). Ten animals per condition were recorded at each timepoint. Recordings were censored if the animal moved out of frame. To calculate pumping rate, each video was trimmed to 30 seconds and scored at 0.5x speed for number of pumps. Significance was between age-matched populations was determined in Graphpad Prism 9 using an unpaired t-test with a Welch correction.

**Percentage of population pumping:** The same population set aside for pharyngeal pumping rate were used for the percentage of the population pumping. To calculate the percentage of each population that remained pumping, animals were scored on Days 3, 8, and 13 of adulthood. At each timepoint, total animals were counted, then scored as either dead, alive and pumping, or alive and not pumping. Animals were scored as alive and not pumping after observation for at least ten seconds and after gentle stroking with a pick. If the individual still failed to move, it was scored as dead and not included in the assay. To determine the percentage of the population pumping, pumping animals were divided by the total number of living animals. Significance was calculated between age-matched and generation-matched populations using a Fisher’s exact test.

## Reagents

Strains were provided by the *Caenorhabditis* Genetics Center (CGC), which is funded by the NIH Office of Research Infrastructure Programs (P40 OD010440). Strains used are indicated in the table below.

**Table:** 

**strain**	**genotype**	**source**
N2	wild-type (Bristol isolate)	CGC
RB1826	*jhdm-1 ( ok2364 ) III *	CGC
